# A Simple, Versatile and Sensitive Cell-Based Assay for Prions from Various Species

**DOI:** 10.1371/journal.pone.0020563

**Published:** 2011-05-31

**Authors:** Zaira E. Arellano-Anaya, Jimmy Savistchenko, Jacinthe Mathey, Alvina Huor, Caroline Lacroux, Olivier Andréoletti, Didier Vilette

**Affiliations:** UMR INRA (Institut National de la Recherche Agronomique) ENVT (Ecole Nationale Vétérinaire de Toulouse) 1225, Interactions Hôte Agent Pathogène, Toulouse, France; Creighton University, United States of America

## Abstract

Detection and quantification of prion infectivity is a crucial step for various fundamental and applied aspects of prion research. Identification of cell lines highly sensitive to prion infection led to the development of cell-based titration procedures aiming at replacing animal bioassays, usually performed in mice or hamsters. However, most of these cell lines are only permissive to mouse-adapted prions strains and do not allow titration of prions from other species. In this study, we show that epithelial RK13, a cell line permissive to mouse and bank vole prion strains and to natural prion agents from sheep and cervids, enables a robust and sensitive detection of mouse and ovine-derived prions. Importantly, the cell culture work is strongly reduced as the RK13 cell assay procedure designed here does not require subcultivation of the inoculated cultures. We also show that prions effectively bind to culture plastic vessel and are quantitatively detected by the cell assay. The possibility to easily quantify a wider range of prions, including rodent experimental strains but also natural agents from sheep and cervids, should prompt the spread of cell assays for routine prion titration and lead to valuable information in fundamental and applied studies.

## Introduction

Transmissible spongiform encephalopathies, or prion diseases, are fatal degenerative disorders of the central nervous system affecting both humans and animals. They are characterized by deposits of abnormal conformers of the PrP protein and the most widely accepted model of prion multiplication is that abnormally-folded PrP recruits and converts the host PrP into new abnormal forms [1], [2], [3]. Abnormal PrP is usually isolated from infected cells and tissues as a detergent-insoluble, protease-resistant, infectious form, named PrP^res^ [4], [5]. However, accumulating evidence has recently highlighted the presence of other abnormal PrP forms beside large PrP^res^ aggregates [6], [7], [8], [9], [10], [11], [12], [13], [14], some of which may also be infectious.

The gold standard for detection and quantification of prion infectivity relies on inoculation of appropriate animals and determination of the time to terminal disease (incubation period method) or the dilution at which a given sample transmits disease to 50% of the inoculated animals (endpoint titration method). Over more than 3 decades, a huge number of titrations have been carried out and provided invaluable information in many aspects of prion research [15], *e.g.*, tissue distribution of prions. Yet bioassays are slow, rather imprecise, costly and require dedicated facilities and large numbers of animals. A major breakthrough was introduced by Weissmann and collaborators showing that some murine prion strains could be titrated in a cell-based assay named Standard Scrapie Cell Assay (SSCA) [16]. The SSCA uses neuroblastoma N2a subclones highly sensitive to 22L and RML strains of mouse prions. In this paradigm, samples to be titrated are incubated with N2a cells grown in microtiter plates. To allow prions to multiply to a biochemically detectable level, all individual inoculated cultures are subpassaged at least 3 times. At the end of the assay, individual PrP^res^-containing cells are immunodetected and automatically enumerated. The SSCA is at least as sensitive as bioassay, cheap, rapid and robust. Since then, cell-based titrations have been instrumental in various aspects of prion research, including the study and the evolution of prion strains [17], [18], their kinetics of multiplication [19], their persistence in the environment [20], the effectiveness of prion decontamination/removal procedures [21], [22], the spontaneous generation of prions [23] and infectivity fractionation [10], [24], [25]. However, applicability is restricted by the limited number of prion strains that can be successfully assayed in N2a cells and by the time-consuming cell culture work, even if SSCA may be suitable to automation.

In strong contrast to N2a cells that are only susceptible to mouse prion strains, RK13 cells expressing the appropriate PrP protein can propagate prions strains from different species [26]. Genetically engineered RK13 cells are permissive to several murine strains (22L, Chandler, Fukuoka-1, RML, MU-02) [27], [28], [29], to a bank vole strain [27] and to natural prion agents from sheep scrapie [30], [31] and Chronic Wasting Disease [32]. Using sheep and mouse prions, we investigated whether RK13 cells are sensitive enough for cell-based detection assays of prion infectivity. We report on an experimental procedure that allows a simple, versatile, robust and sensitive detection of prions. More specifically, the cell culture work is strongly reduced compared with all other prion cell assays as RK13 cultures do not require to be subpassaged after challenge. Interestingly, we also show that infectivity is efficiently bound by cell culture plastic and quantitatively detected by our cell assay.

## Results

Previous studies demonstrated that expression of ovine, elk, mouse or bank vole PrP in the RK13 cell line renders the resulting ovRK13, elRK13, moRK13 or voRK13 cells permissive to prions of the corresponding species, as assessed by accumulation of PrP^res^ and infectivity in the infected cultures [26]. By using doxycycline (dox)-regulatable PrP constructs, no detectable PrP^res^ accumulates when the cells do not express the transfected PrP, *i.e.*,when the experiments are performed in the absence of dox [27], [30], [31].

### OvRK13 cell assay of sheep prions

To determine the sensitivity of the RK13 cell assay to sheep prion infection, we used PG127, a natural sheep scrapie isolate [33] propagated in *tg338* transgenic mice expressing the ovine PrP [34]. Infectivity of PG127 10% brain homogenate was assessed by intracerebral inoculation of 10-fold dilutions into *tg338* mice. The last dilution inducing clinical disease in 100% of the inoculated animals (n = 6) was 10^−5^ (the incubation period was 106 days ±6 days, mean ± SD and the calculated infectious titer was 10^7.7^ LD_50_/ml).

**Figure 1:**
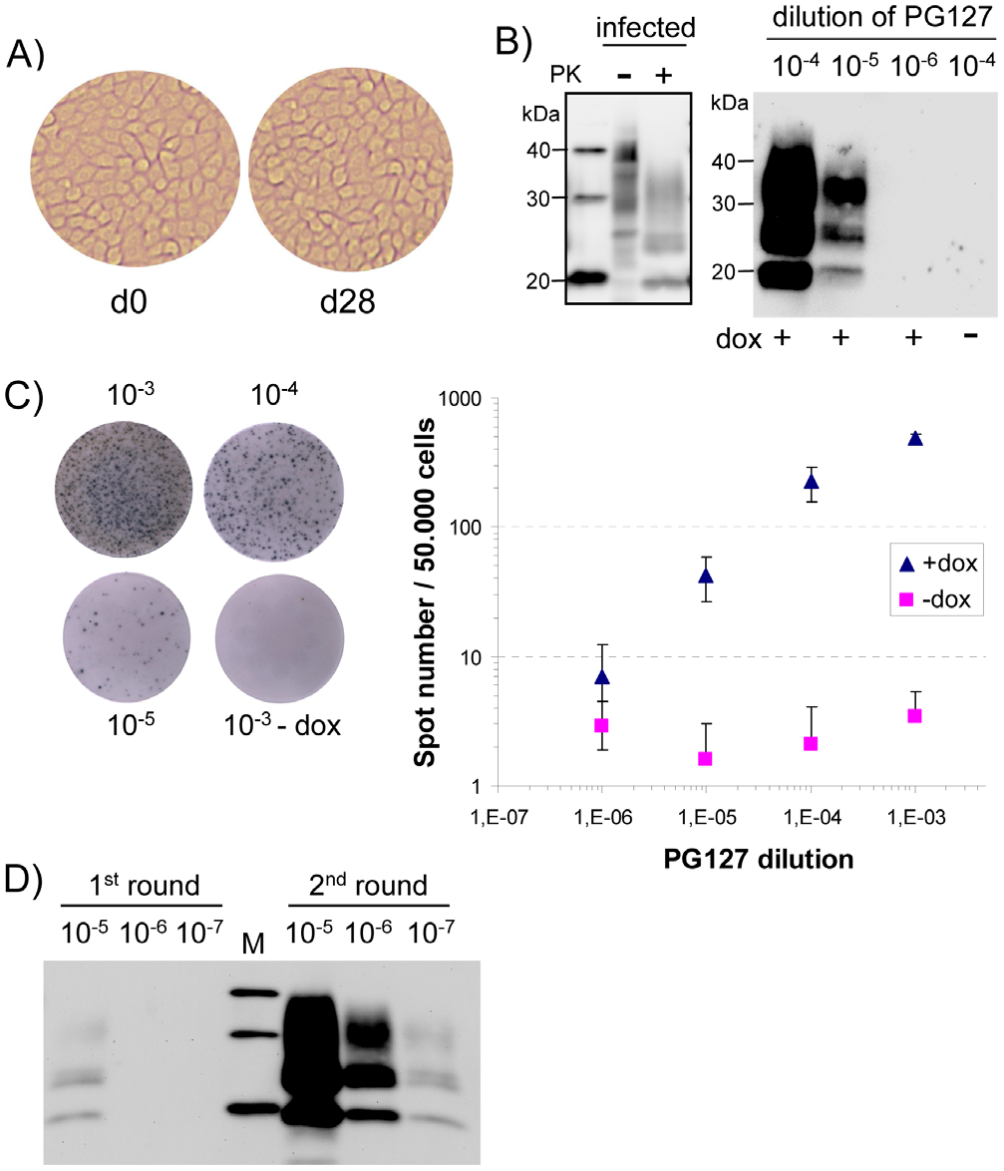
Sensitivity of ovRK13 cell assay for the detection of PG127 ovine prion. A) Morphology of inoculated ovRK13 cultures kept in the same wells during the whole cell assay procedure (d0 is the time of inoculation and d28 is 4 weeks later). B) Sensitivity of ovRK13 cell assay as assessed by immunoblotting. *Right panel:* Serial 10-fold dilutions (from 10^−4^ to 10^−6^) of infectious PG127 10% brain homogenate were inoculated to single wells of ovRK13 cells. Four weeks later, inoculated cultures were analyzed for PrP^res^ by immunoblotting. Positive transmission was detected for dilutions up to 10^−5^. No PrP^res^ was observed when inoculated ovRK13 did not express the ovine PrP (dox-). *Left panel:* total PrP from infected cells was analyzed before (−) or after (+) PK digestion to illustrate band shift upon PK proteolysis. M are standard molecular mass marker proteins (20, 30 and 40 kDa). C) Sensitivity of ovRK13 cell assay as assessed by Elispot. Replicate wells from the same experiment shown in Fig. 1B were analyzed. Left: representative wells of an Elispot plate showing spots given by ovRK13 cells exposed to the indicated dilutions of PG127. Right: double-logarithmic plot of spot number versus PG127 dilution shown for inoculations in the presence (triangle) or in the absence (square) of dox. For each dilution, the mean value ± SD of 8 measurements is shown. Background values for ovRK13 cells inoculated in the absence of dox are less than 4 spots per 50,000 cells. D) Sensitivity is strongly improved by 2 successive rounds of cell assay. Serial 10-fold dilutions (from 10^−5^ to 10^−7^) of infectious PG127 10% brain homogenate were inoculated to duplicate wells of ovRK13 cells. Four weeks later, PrP^res^ in a 1^st^ set of inoculated cultures was isolated (1^st^ round) while cultures of the 2^nd^ set were homogenized to inoculate new ovRK13 cells. Four weeks later, PrP^res^ was isolated (2^nd^ round) and all the samples were analyzed by immunoblotting. M are standard molecular mass marker proteins (20, 30 and 40 kDa).

This PG127 10% brain homogenate was serially diluted in cell culture medium and 10-fold dilutions (from 10^−4^ to 10^−6^) were applied to confluent ovRK13 cells in 6-well plates (one well per dilution). One week later, the infected media were removed and replaced by fresh medium. RK13 cells, like many other epithelial cell lines [35] can be kept for weeks as viable, confluent monolayers of polarized cells ([36]–[37] and Fig. 1A). The inoculated ovRK13 cultures were kept for 3 more weeks in the same wells (the medium being changed once a week) and detergent-solubilized, digested with proteinase K (PK) and assayed for PrP^res^ by immunoblotting. Transmission was scored positive when a typical PrP^res^ signal (Fig. 1B, left) was observed. Data from 16 independent transmissions carried out by 4 different operators over a 6 month-period demonstrated positive transmission for all cultures inoculated with PG127 dilutions down to 10^−5^. No detectable PrP^res^ was observed in cultures inoculated with 10^−6^ dilutions. A representative immunoblot is shown in Fig. 1B, right. When the experiments were performed in the absence of dox, the inoculated cultures were PrP^res^ negative. This important control demonstrates that the detected PrP^res^ indeed results from *de novo* infection of the cells and not from residual PrP^res^ from the diluted inocula. In addition, immunoblotting was not sensitive enough to detect the very low amounts of PrP^res^ in the 10^−5^ dilutions (data not shown).

We also determined the sensitivity of our cell assay by analyzing the number of PrP^res^-containing cells in the inoculated cultures. In this assay, 50,000 cells from the same experiment shown in Fig. 1B were filtered onto membranes of Elispot microtiter plates, the cells were then lyzed, PK-digested and PrP^res^ was immunodetected after guanidine hydrochloride (GdnHCl) denaturation. The resulting spots, representing individual PrP^res^-containing cells, are depicted in Fig. 1C, (left) and were automatically counted with an imaging equipment. Double-logarithmic plots of PG127 dilution values *versus* detected spots are shown in Fig. 1C, (right). In our conditions, the sensitivity of detection by Elispot was similar to that by immunoblotting, *i.e.*, positive transmission was observed for a 10^−5^ dilution of PG127 (42±16 spots per 50,000 cells) and spot number was linear between two logs of dilutions. Consistent with immunoblot analysis (Fig. 1B), PrP^res^ from PG127 inoculum diluted beyond 10^−3^ was not detected in inoculated, non dox-treated cultures. Although Elispot and immunoblotting were equally sensitive for detecting infected cultures in our experimental conditions, these two techniques have distinct characteristics that must be considered for their use in cell-based assays. The whole Elispot procedure, including trypsinization, filtration, immunolabelling of cells and counting is suitable to automatisation, which is a major advantage for large scale titrations. Immunoblot detection is less rapid and more labor intensive but does not rely on sophisticated equipments and allows analytical assessment of PrP^res^.

In cultures infected with low doses of inoculum, a very small number of cells has detectable PrP^res^ amounts (42 cells ±16 cells out of 50,000 cells for 10^−5^ dilution of PG127), suggesting that infectivity is clustered in a very small number of highly infected cells. We thought that physical disruption of these cells would redistribute the prions, allowing infection of more cells during a second round of cell-based assay. To test this possibility, duplicate cultures of ovRK13 were inoculated, one series was lyzed one month post-challenge while cells from each well from the 2^nd^ series was homogenized and used to inoculate new ovRK13 cells for a 2^nd^ round of cell assay. PrP^res^ levels after one or two rounds were analyzed by immunoblot. Typical results (Fig. 1D) revealed that inoculated cultures that appear negative in the first round (10^−6^ and 10^−7^) contained infectivity that was detected upon a 2^nd^ round of cell assay. Thus, performing two successive rounds dramatically improved (by 100-fold) the cell assay sensitivity. Infectivity in 10^−7^ dilutions, not detected upon inoculation into *tg338* mice, was clearly evidenced through the ovRK13 cell assay.

### MoRK13 cell assay of mouse RML prions

**Figure 2:**
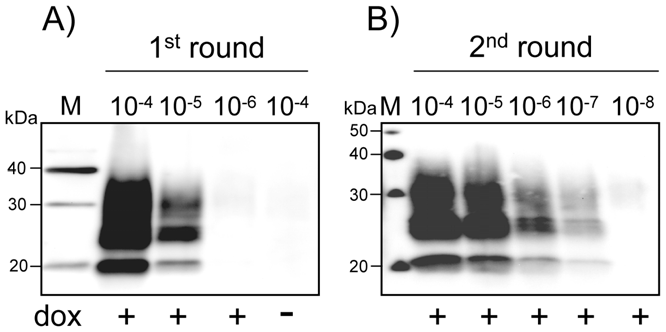
Sensitivity of moRK13 cell assay for the detection of RML mouse prions. Serial 10-fold dilutions (from 10^−4^ to 10^−8^) of RML 10% brain homogenate were inoculated in duplicate to moRK13 cells and the inoculated cultures were proceeded as in figure 1D. PrP^res^ was analyzed by western blot after one (A) or two rounds (B) of cell assay. M are standard molecular mass marker proteins in kDa.

To determine the sensitivity of the RK13-based assay for prion strains from other species, we chose RML, widely used in the mouse scrapie model. Serial 10-fold dilutions of RML 10% brain homogenate inoculated into *tg20* mice showed that the last dilution inducing clinical disease in all mice (n = 6) was 10^−6^ (the incubation period was 108 days ±6 days, mean ± SD). Serial 10-fold dilutions of this RML 10% brain homogenate (from 10^−4^ to 10^−8^) were inoculated to moRK13 cells. The cultures were solubilized 4 weeks later, PK-digested and analyzed for the presence of PrP^res^. Out of 8 independent experiments, all cultures inoculated with 10^−5^ were PrP^res^ positive (a typical result is shown in Fig. 2A). In two experiments, a PrP^res^ signal was also detected in cells inoculated with the 10^−6^ dilution of RML. As observed with ovine prions, a second round of cell assay increased the sensitivity of RML detection by 100-fold (Fig. 2B), *i.e.*, inoculated cultures that appear negative in the first round (10^−6^ and 10^−7^) contained infectivity that was detected upon a 2^nd^ round of cell assay. Thus, 10^−7^ dilutions that do not transmit disease to *tg20* mice lead to a detectable infection of moRK13 cultures.

### Cell assay of prions bound to plastic surface

**Figure 3:**
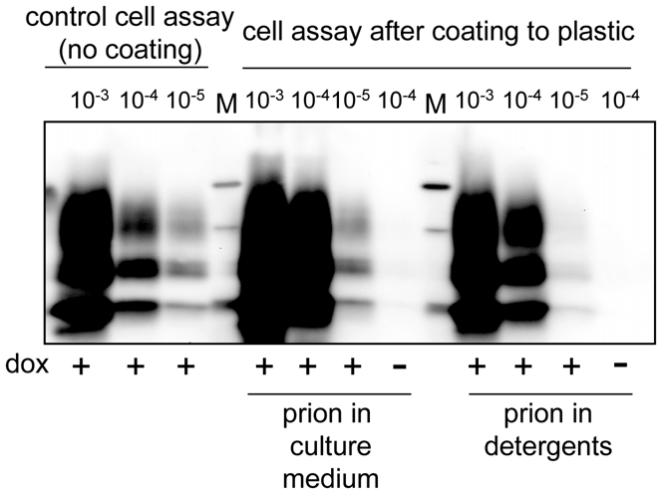
Cell assay detection of plastic-bound prions. Infectivity from 10% PG127 brain homogenate was diluted either in culture medium (prion in culture medium) or in Triton-DOC lysis buffer (prion in detergents) and incubated for 2 h into plastic wells. Samples were removed, wells were thoroughly rinsed and air-dried. OvRK13 cells were then seeded in the presence (+) or in the absence (−) of dox. PrP^res^ in the cultures was analyzed 4 weeks later by immunoblotting and compared to PrP^res^ levels in ovRK13 cultures subjected to the standard cell assay (no coating). M are standard molecular mass marker proteins (20, 30 and 40 kDa).

Prions strongly bind to stainless steel wires and the contaminated wires efficiently transmit disease to mice [38], [39], [40] and infection to recipient cultured target cells [21]. Prion-coated wires, as models of contaminated surgical metal devices, were used to assess the effectiveness of decontamination procedures [38], [39], [41] [21] [42] [43], [44]. The propensity of prions for steel surface binding led recently to a blood-based assay of vCJD [45]. There is also suggestion that prions bound to tissue culture plastic may elicit infection of seeded cultured cells [46]. To further examine this possibility, the 10% PG127 homogenate was diluted in complete culture medium and serial 10-fold dilutions were deposited for 2 h in tissue culture plastic wells. Since it would be also useful to detect prion infectivity in detergents buffers used in cell or tissue fractionation [6], [10] or in PMCA reactions [47], [48], the 10% PG127 was solubilized, serially diluted in Triton-DOC lysis buffer and deposited in plastic wells as well. The samples were then removed, the wells thoroughly washed with PBS and air-dried for 2 h. OvRK13 cells were seeded into these wells and infection was allowed to proceed for 4 weeks. We found (representative data from 4 experiments are shown in Fig. 3) that infection of cultures seeded in prion-coated wells was as efficient as that observed with the standard procedure, *i.e.*, cultures seeded in 10^−5^-coated wells were PrP^res^ positive. Interestingly, coating and cell detection of detergent-solubilized prions were also very effective. These findings indicate that prions can be quantitatively detected by our cell assay upon binding to plastic surfaces.

## Discussion

The cell-based assay designed in this study allows a simple, versatile, rapid, robust and sensitive detection of ovine-derived and mouse RML prions. Since RK13 cells are permissive to several natural sheep scrapie isolates [30], [31], to 22L, Chandler, Fukuoka-1, RML and MU-02 strains of mouse prions [27], [28], [29], to a strain of bank vole prions [27], the procedure described here should be useful for an easy and sensitive detection of a wide range of prions. Notably, it should improve and/or facilitate the recently reported RK13-based titration of Chronic Wasting Disease agent [32].Performing two successive rounds of infection increased the sensitivity of the ovRK13 and moRK13 cell-based assays by a 100-fold, allowing detection of infectivity in 10^−7^ dilutions of 10% PG127 and RML homogenates, albeit at the cost of prolonging the assay time. In that case, sensitivity of the RK13 cell assay appears similar to that of the mouse bioassay. The amount of 10% PG127 or RML brain homogenates to infect ovRK13 or moRK13 cells, respectively, was 3 10^−4^ µl (3,000 µl ×10^−7^). This is very similar to the lower amount of 10% PG127 or RML that transmits disease to 100% of *tg338* or *tg20* mice, *i.e.,* 2 10^−4^ µl (20 µl ×10^−5^). Considering that 3,000 µl of PG127 at a 10^−5^ dilution resulted in 42 spots (cell units), there are about 1.3 10^6^ cell units/ml of 10% PG127. Since a second round increases the sensitivity by about 100-fold, we estimate that 10% PG127 contains about 1.3 10^8^ cell units/ml, a value closed to the titer obtained by *tg338* bioassay (10^7.7^ LD50/ml). The accurate comparison of moRK13 sensitivity to that of other cell lines widely used for mouse prion titration (*e.g.*, PK1, CAD5, LD9) would require the use of the same RML preparations. However, these cell lines led to a detectable infection for RML dilutions up to 10^−5^–10^−6^ (10^−6^–10^−7^ in the designation’s authors as their 10% RML brain homogenate was set as the 10^−1^ dilution) [18], [49]. This suggests that moRK13 cells have a sensitivity roughly similar to these lines.

In sharp contrast to the other cell lines previously used to titrate mouse [18] and sheep prions [50], no subcultivation of inoculated RK13 epithelial cultures is required before analysis. This unique feature is a crucial operational advantage as serial passaging of inoculated cultures is very time-consuming and costly, especially when assaying large numbers of samples. For example, inoculated MovS cultures [51] have to be passaged 8 times for optimal PG127 detection [50]. In addition, the slow down of cell division may participate in high sensitivity of the assay as cell division was reported to decrease steady state levels of PrP^res^ [52]. In contrast with N2a and other sublines whose susceptibility is not preserved during prolonged subcultivation [18], we did not observe any decline in sensitivity when ovRK13 and moRK13 cells were serially passaged for six months (*i.e.*, 24 serial 1:4 splits, longer periods of subcultivation were not systematically explored). Such stability contributes to the ease-of-use of the RK13 cell assay.

The binding of prions onto a plastic surface is a further example of prion avidity for various surfaces including steel [38], [40] and soil particles [53]. This amazing propensity of prions to bind solid surfaces and/or particles is an efficient and inexpensive mean to concentrate prions as illustrated by the detection of steel-bound vCJD blood prions [45]. How prions adsorb to these surfaces is unknown but there is compelling evidence that bound prions remain infectious [20], [21], [38], [54]. Further studies are underway to establish whether small amounts of prions in biological fluids can be concentrated onto plastic surface and be detected by our cell assay.

Over the recent past years, cell-based assays have proven to be valuable tools for prion detection and titration. We believe that our easy to handle and labor non intensive RK13-based assay will further contribute to routine detection of prions and may help to substitute cultured cells for animal bioassay in various aspects of prion research. In addition, the remarkable binding property of prions to tissue culture plastic should allow routine detection of prion infectivity in samples that are toxic for cells, including those from PMCA reactions.

## Materials And Methods

### Ethics statement

All animal experiments were performed in compliance with our institutional and national guidelines, in accordance with the European Community Council Directive 86/609/EEC. The experimental protocols were approved by the INRA Toulouse/ENVT ethics committees, and were performed in the approved animal facilities (C 31 555 27) of author’s institution by an agreed experimentator (311055503).

### Cells

OvRK13 cells expressing the ovine PrP protein (the Rov9 clone) have been previously described [31]. We generated moRK13 cells expressing the mouse PrP (allele a) as described previously [27] and the cell clone used in this study was mo1. The cells were maintained at 37°C in 5% CO2 in Opti-MEM medium (Invitrogen) supplemented with 10% fetal bovine serum, 100 U/ml penicillin, and 10 µg/ml streptomycin. To induce the expression of ovine or mouse PrP, 1 µg/ml doxycycline (Sigma-Aldrich) was added to the culture medium.<

### Prion agents

The sheep isolate (PG127)[33], propagated once in sheep, was amplified in *tg338* mice [34] and prepared as described previously [6]. The mouse RML strain was propagated in C57BL/6 mice. At the terminal stage, brains were homogenized at 10% (wt/vol) in 5% sterile glucose using a high-speed homogenizer (TeSeE Precess 48 system).

### Cell-based assay procedure

OvRK13 or moRK13 cells were seeded in six-well plates (TPP) with or without doxycycline treatment. When confluent, the cultures were exposed to 3 ml of culture medium containing serial 10-fold dilutions of the PG127 or RML 10% infectious brain homogenates. One week later, the media were renewed. Infection was allowed to proceed for 3 more weeks with one medium change per week. At the end, the cells were either solubilized for immunoblot analysis of PrP^res^ or processed for Elispot detection of PrP^res^-containing cells. Alternatively, the cells were trypsinized, the cell pellets from each well were resuspended in 500 µl of 5% sterile glucose and were homogenized with the high-speed homogenizer. Half of the resulting cell extracts was used to inoculate ovRK13 or moRK13 cells for a second round of cell assay.

### Coating of prions to plastic

Threeµl of 10% PG127 brain homogenate (this corresponds to the amount of PG127 in 3 ml of a 10^−3^ dilution) were diluted into 250 µl of culture medium and then serially diluted 10-fold into 250 µl of culture medium. Alternatively, the same amount of PG127 was diluted into 250 µl of normal sheep brain proteins (1 mg/ml) solubilized in Triton-DOC lysis buffer and further diluted 10-fold into the same buffer. All the samples (250 µl) were incubated into pre-wet polystyrene wells of six-well plates (TPP) at room temperature with occasional agitation. Two hours later, the samples were removed, the wells were washed twice with 3 ml of PBS and air dried for 2 h. OvRK13 cells (300,000 cells in 3 ml of culture medium with or without dox, as indicated) were seeded into the wells and the cultures were kept for 4 weeks with one medium change every week.

### Immunoblotting detection of PrP^res^ in inoculated cultures

Cell cultures were rinsed with cold PBS and solubilized for 10 min at 4°C in Triton-DOC lysis buffer (50 mM Tris/HCl (pH 7.4), 0.5% Triton-X100, 0.5% sodium deoxycholate). The lysates were clarified by low speed centrifugation (425 X *g*, 1 min) and cellular proteins in the post-nuclear supernatants were quantified by bicinchoninic acid (BCA, Pierce). Digestion of 750 µg – 1,000 µg of proteins with PK (recombinant grade, Roche) was performed for 2 h at 37°C with a mass ratio of 4 µg of PK per mg of cellular proteins and the reaction was stopped by addition of Pefabloc (Sigma-Aldrich) to 4 mM. PK-digested samples were centrifuged for 30 min at 20,000 X *g* and pellets were analyzed by western blot. Samples separated by 12% SDS-PAGE electrophoresis were transferred to PVDF membranes (Bio-Rad). The western blots were stained for PrP with Sha31 mAb [55]. Filters were developed using an ECL+ reagent kit (Amersham-GE Healthcare) and visualized with a Bio-Rad VersaDoc imaging system.

### Elispot detection of infected cells

The procedure has been described in detail [56]. Trypsinized cells were resuspended in PBS and 50,000 cells were aspirated into each well of a Multiscreen 96-well filter plate (Millipore). The plate was incubated at 50°C for 1 h. Dried wells were incubated for 30 min at 37°C with 20 µg/ml of PK in Triton-DOC lysis buffer and digestion was stopped with 4 mM Pefabloc. Proteins were denatured with GdnHCl 8 M in PBS for 10 min. Filters were saturated for 1 h with 0.5% BSA in tris-buffered saline with tween20 (TBST), incubated with Sha31 mAb and then with peroxidase-coupled secondary antibody. Bound antibodies were visualized with TMB stabilized substrate for horseradish peroxidase (Promega) and spots were detected and enumerated with an Elispot reader system (AID GmbH).

### Mice bioassays

10% PG127 or 10% RML brain homogenates were serially 10-fold diluted into Opti-MEM medium supplemented with 10% fetal bovine serum, 100 U/ml penicillin, and 10 µg/ml streptomycin, and 20 µl were inoculated intracerebrally into *tg338* [34] or *tg20* [57] mice, respectively. Mice were then clinically monitored until the occurrence of scrapie clinical signs, at which time they were euthanized.
